# Association between alcohol consumption and oesophageal microbiota in oesophageal squamous cell carcinoma

**DOI:** 10.1186/s12866-021-02137-x

**Published:** 2021-03-06

**Authors:** Wenqing Rao, Zheng Lin, Shuang Liu, Zhihui Zhang, Qianwen Xie, Huilin Chen, Xi Lin, Yuanmei Chen, Huimin Yang, Kaili Yu, Zhijian Hu

**Affiliations:** 1 Department of Epidemiology and Health Statistics, Fujian Medical University Fujian Provincial Key Laboratory of Environment Factors and Cancer, School of Public Health, Fujian Medical University, Fuzhou, 350122 China; 2 Department of Radiation Oncology, Anxi County Hospital, Quanzhou, 352400 China; 3 Department of Statistics Office, Zhangzhou Affiliated Hospital of Fujian Medical University, Zhangzhou, 363000 China; 4 Department of Thoracic Surgery, Fujian Provincial Cancer Hospital Affiliation to Fujian Medical University, Fuzhou, 350014 China; 5 Key Laboratory of Ministry of Education for Gastrointestinal Cancer, Fujian Medical University, Fuzhou, 350122 China

**Keywords:** Oesophageal squamous cell carcinoma (ESCC), Alcohol, Microbiota

## Abstract

**Background:**

Microbiota has been reported to play a role in cancer patients. Nevertheless, little is known about the association between alcohol consumption and resultant changes in the diversity and composition of oesophageal microbiota in oesophageal squamous cell carcinoma (ESCC).

**Methods:**

We performed a hospital-based retrospective study of 120 patients with pathologically diagnosed primary ESCC. The relevant information for all study participants were collected through a detailed questionnaire. The differences in adjacent tissues between non-drinkers and drinkers were explored using 16S rRNA gene sequencing. Raw sequencing data were imported into QIIME 2 to analyse the diversity and abundance of microbiota. Linear discriminant analysis effect size (LEfSe) and unconditional logistic regression were performed to determine the bacterial taxa that were associated with drinking.

**Results:**

The Shannon diversity index and Bray-Curtis distance of oesophageal microbiota were significantly different among drinkers(*P* < 0.05). The alcohol-related bacteria were primarily from the orders Clostridiales, Gemellales and Pasteurellales, family Clostridiaceae, Lanchnospiraceae, Helicobacteraceae, Alcaligenaceae, Bacteroidaceae, Pasteurellaceae and Gemellaceae*;* genus *Clostridium, Helicobacter*, *Catonella, Bacteroides, Bacillus*, *Moraxella,* and *Bulleidia;* and species *B. moorei and longum* (genus *Bifidobacterium*)*.* In addition, the diversity and abundance of these microbiota were observed to be affected by the age, residential districts of the patients, and sampling seasons. Moreover, the higher the frequency and years of alcohol consumption, the lower was the relative abundance of genus *Catonella* that was observed.

**Conclusion:**

Alcohol consumption is associated with alterations in both the diversity and composition the of the oesophageal microbiota in ESCC patients.

**Supplementary Information:**

The online version contains supplementary material available at 10.1186/s12866-021-02137-x.

## Background

Cancer is generally regarded among one of the dominant causes of death in recent times. The incidence rate of oesophageal cancer (EC) ranks seventh in the world, while the mortality rate ranks sixth worldwide [1]. According to the cancer statistics for 2015 in China, the EC incidence ranked third in the country, with ~ 477,900 cases, the corresponding mortality was reported to be ~ 375,000 cases [2]. There are two main histological type of EC— the oesophageal squamous cell carcinoma (ESCC) and the oesophageal adenocarcinoma — of which, ESCC has been confirmed as the predominant histological subtype in China [1]. Smoking, alcohol abuse, consumption of very hot beverages and susceptibility in genetic loci have also been identified as potential risk factors for ESCC [3]. However, in spite of the remarkable development in the diagnosis and treatment of ESCC, the 5-year survival rate has not significantly improved [4]. Therefore, certain other risk factors underlying the incidence of ESCC also need to be investigated.

The microbiome is receiving a great deal of attention in recent years given its influence on human diseases including malignancy [5]. Millions of microbes are known to constantly interact with the host within the human system such as the gastrointestinal tract [6]. Both host physiology and environmental factors can cause significant alterations of the microbiota [7]. In fact, alcohol is a known disruptor of microbes and quite a few studies have investigated the effects of alcohol on the microbiota in animals and humans [8, 9].

Rezasoltani confirmed that mucosal microbiota might be more stable and specific to different stages of colorectal cancer [10]. The oesophageal microbiota in ESCC, however, have not been studied extensively till date, and the existing reports either primarily described the characteristics of the oesophageal microbiota, or found some prognosis-related bacteria in ESCC. For instance, specific microbial communities were detected in the oesophagus of patients with early stage ESCC and oesophageal squamous dysplasia (the precursor of ESCC), compared to the general microbial communities detected in a healthy oesophagus [11]. Moreover, Shao [12] described the microbes of paired tumour and non-tumour samples from ESCC patients, while, Liu [13] identified the impact of oesophageal microbiota on ESCC progression. Thus, it is generally assumed that the microbiota in the oesophagus may contribute to the development of ESCC. However, relationships between environmental risk factors and altered oesophageal microbiota in ESCC have not been explored previously.

Therefore, in this study, we aim to investigate the association between alcohol consumption and the resulting alterations in the diversity and composition of oesophageal microbiota among ESCC patients of different age groups from different districts, during different sampling seasons, in an attempt to determine whether alcohol consumption frequency and years, play a significant role in the alteration of oesophageal microbiota, and thereby, to elucidate the relationship between alcohol consumption and oesophageal microbiota in patients with ESCC.

## Results

### Participant characteristics

The major demographic and baseline clinical features of drinkers and non-drinkers were analysed and are presented in Table 1. In our study, 60 patients (50.0%) were non-drinkers and 60 (50.0%) were drinkers. There was no significant difference between the drinkers and non-drinkers with respect to age, residential district, sampling season, tumour location, T stage, N stage, stage and differentiation; however, the distribution based on gender, smoke, and tea consumption varied significantly (all *P* < 0.001).

**Table 1:** Major demographic and baseline clinical features

Characteristics	Non-drinkers	Drinkers	***χ2***	***P***
Gender			31.707	< 0.001
Female	29 (48.3%)	2 (3.3%)		
Male	31 (51.7%)	58 (96.7%)		
Age			1.205	0.272
≤ 60 years	25 (41.7%)	31 (51.7%)		
> 60 years	35 (58.3%)	29 (48.3%)		
Smoke			34.66	< 0.001
No	34 (56.7%)	4 (6.7%)		
Yes	26 (43.3%)	56 (93.3%)		
Tea			13.807	< 0.001
No	25 (41.7%)	7 (11.7%)		
Yes	35 (58.3%)	53 (88.3%)		
Residential distract			3.429	0.064
Zhangzhou	30 (50.0%)	20 (33.3%)		
Others^a^	30 (50.0%)	40 (66.7%)		
Sampling season			2.194	0.139
Winter/Spring	39 (65.0%)	31 (51.7%)		
Summer/Autumn	21 (35.0%)	29 (48.3%)		
Tumour location			0.185	0.911
Upper	5 (8.3%)	6 (10.0%)		
Middle	28 (46.7%)	29 (48.3%)		
Lower	27 (45.0%)	25 (41.7%)		
T stage			0.048	0.827
1/2	13 (21.7%)	14 (23.3%)		
3/4	47 (78.3%)	46 (76.7%)		
N stage			0.036	0.849
0	21 (35.0%)	22 (36.7%)		
≥ 1	39 (65.0%)	38 (63.3%)		
Stage			0.000	1.000
I/II	24 (40.0%)	24 (40.0%)		
III	36 (60.0%)	36 (60.0%)		
Differentiation			1.759	0.485
Well	4 (6.7%)	4 (6.7%)		
Moderately	50 (83.3%)	45 (75.0%)		
Poor	6 (10.0%)	11 (18.3%)		

^a^: other districts of the Fujian province

### Diversity of oesophageal microbiota

**Fig. 1:**
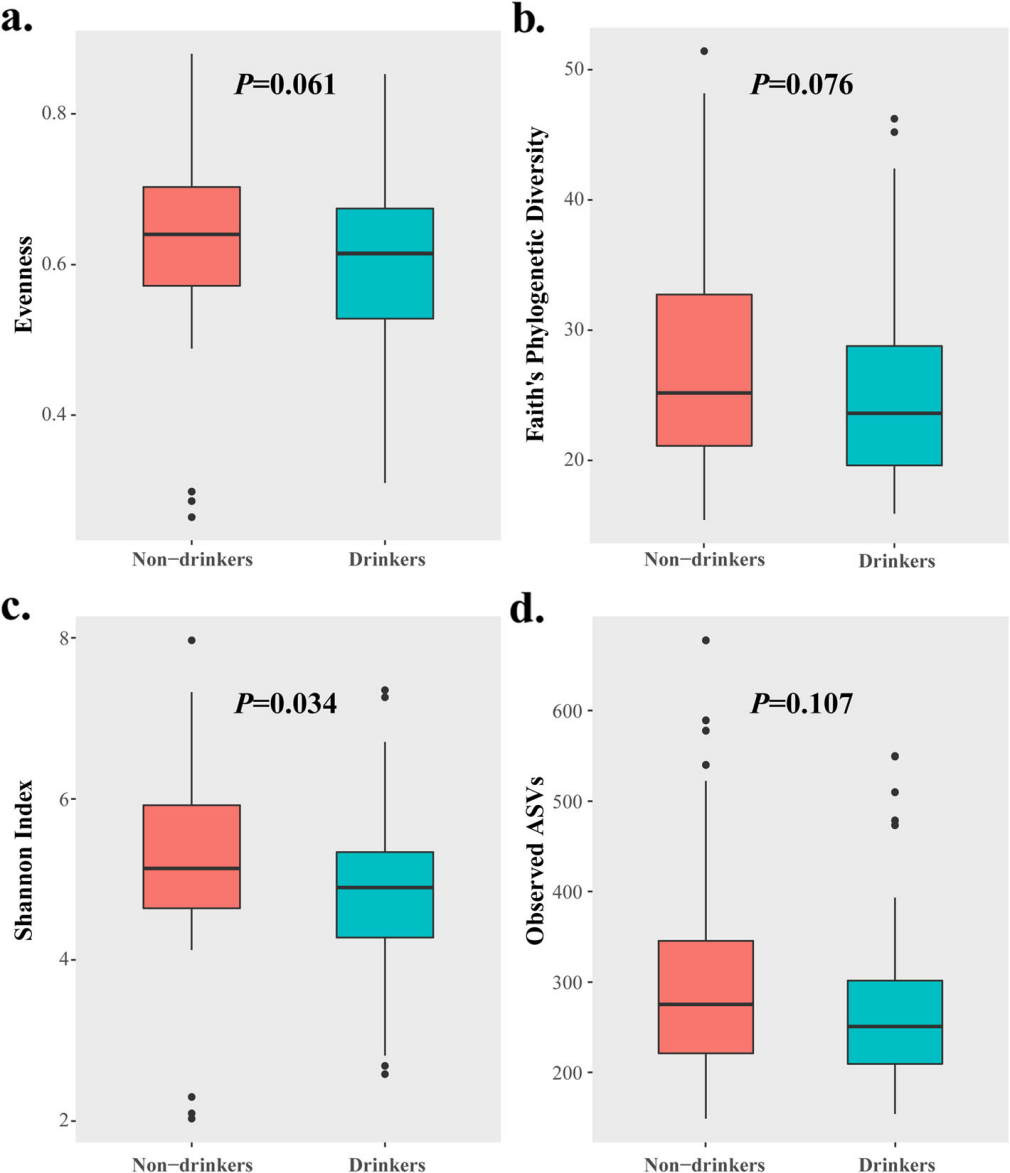
Alpha diversity measures between non-drinkers and drinkers.(**a**: Evenness index; **b**: Faith’s Phylogenetic diversity index; **c**: Shannon diversity index; **d**:Observed ASVs)

The boxplot charts of alpha diversity measures are shown in Fig. 1. There were no significant differences between drinkers and non-drinkers with respect to species evenness, Faith’s phylogenetic diversity, and observed ASVs. The Shannon diversity index of non-drinkers was higher than that of drinkers(*P* = 0.034).

**Fig. 2:**
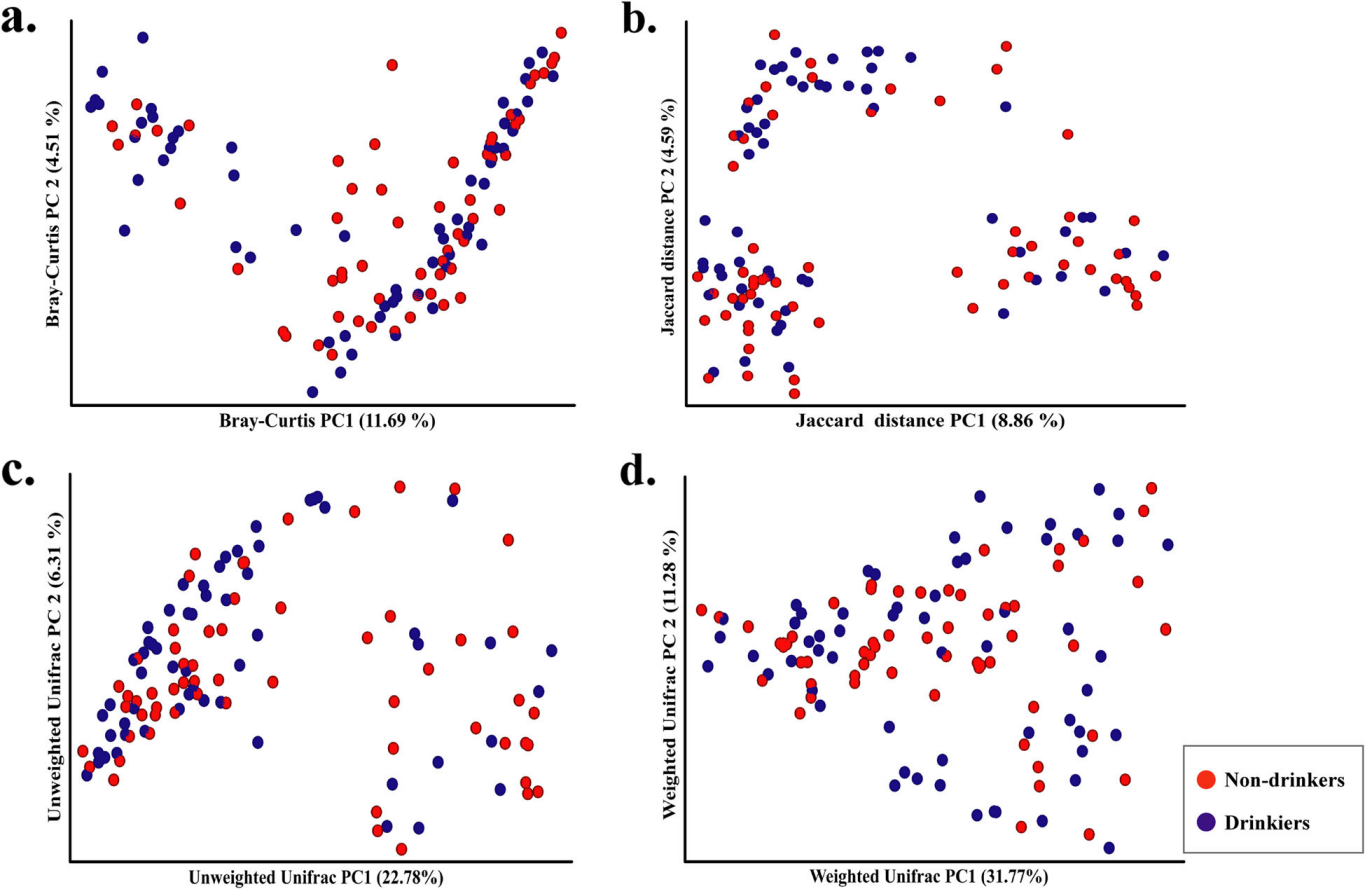
Four distance PCoA data labeled by non-drinkers (red) and drinkers (blue). (**a**: Bray-Curtis distance; **b**: Jaccard distance; **c**: Unweighted Unifrac distance; d: Weighted Unifrac distance)

The principal coordinate analysis (PCoA) was carried out and plotted to compare the microbial communities of non-drinkers and drinkers. The microbial communities of the two groups were observed to be similar (Fig. 2), though the multivariate beta diversity based on Bray-Curtis distance showed significant differences between the two groups (Adonis test, *P* = 0.044; Table 2).

**Table 2:** Beta diversity analysis between non-drinkers and drinkers

Distance	PERMANOVA	Adonis test^**#**^	
		R^**2**^	***P***
Bray-Curtis distance	0.063	0.012	0.044
Jaccard distance	0.102	0.010	0.066
Unweighted UniFrac distance	0.059	0.013	0.050
Weighted UniFrac distance	0.221	0.011	0.209

^#^: Adjusted by gender, age, smoke, tea, season, residential district, hot food, hard food, pickled food, fried food, fruit, vegetable, stage and tumour location

### Taxon abundance analysis

We identified 17 phyla, 30 classes, 54 orders, 96 families, 175 genera, and 239 species of microbes from our 120 samples. We also investigated whether specific taxa differed between the non-drinkers and drinkers.

### Linear discriminant analysis effect size (LEfSe)

**Fig. 3:**
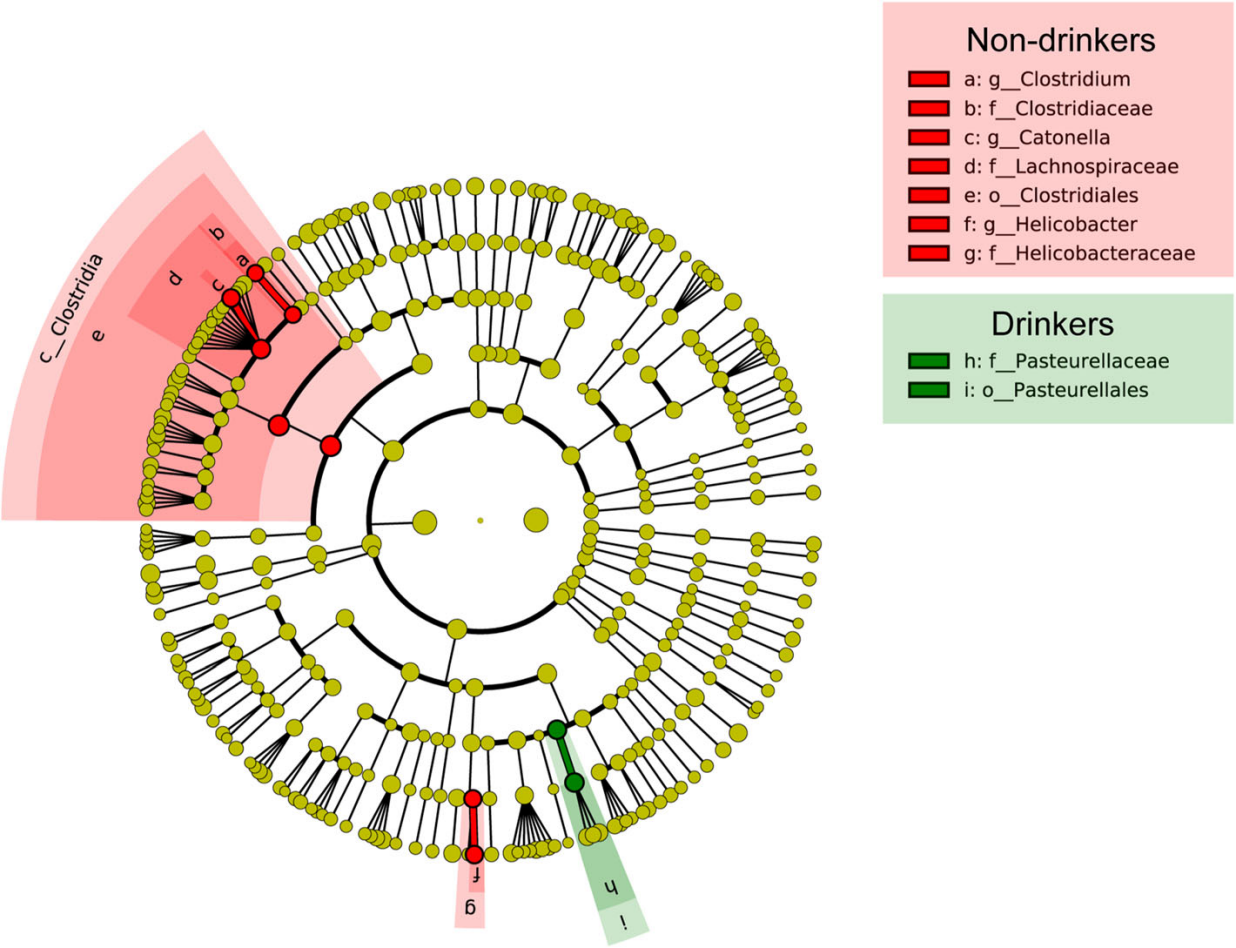
Phylogenetic relationships among specific taxa. (red dots: the high abundance of bacteria in non-drinkers. Green dots: the high abundance of bacteria in drinkers)

Several differences were detected in the abundance of microbiota between the non-drinkers and drinkers. LEfSe suggested nine bacteria that were significantly different between the two groups (Fig. S1). Different bacteria were defined by a linear discriminant analysis (LDA) score cut-off of 2.5. The drinkers showed a higher abundance of microbes from the order Pasteurellales, particularly the family Pasteurellaceae. Conversely, non-drinkers had a higher abundance of order Clostridiales*,* family Clostridiaceae*,* family Lanchnospiraceae, family Helicobacteraceae*,* genus *Clostridium*, genus *Helicobacter* and genus *Catonella*. Phylogenetic relationships among specific taxa are displayed in the cladogram (Fig. 3).

### Unconditional logistic regression analysis

All the detected taxa were divided into dominant and rare microbiota. There were 11 phyla, 18 classes, 27 orders, 49 families, 69 genera and 86 species of dominant microbiota; 6 phyla, 12 classes, 27 orders, 47 families, 106 genera and 153 species of rare microbiota. We then performed unconditional logistic regression analysis to determine association between alcohol consumption and alterations in the oesophageal microbiota.

The overall details revealed through the analysis have been incorporated in Table 3. Among the dominant microbiota, the relative abundance of species *longum* (genus *Bifidobacterium*); the order Gemellales, its family Gemellaceae, genus *Bulleidia,* and its species *B. moorei* were significantly higher in drinkers, while, the relative abundance of the members of family Bacteroidaceae*,* particularly its genus *Bacteroides* and genus *Catonella* (family Lachnospiraceae) were significantly lower in drinkers. Among the rare microbiota, the relative abundance of the genus *Bacillus* and genus *Moraxella* was significantly higher among drinkers, whereas that of the family *Alcaligenaceae* was significantly lower.

**Table 3:** Overall analysis between non-drinkers and drinkers (p:phylum, o:order,f:family, g:genus, s:species)

Bacteria	Univariate analysis			Multivariate analysis*		
	*OR*	95*%CI*	*P*	*OR*	95*%CI*	*P*
**Dominant microbiota**						
*p_Actinobacteria*						
*s_longum*	1.710	0.833–3.548	0.218	3.232	1.159–9.602	0.034
*p_Bacteroidetes*						
*f_Bacteroidaceae*	0.290	0.134–0.607	0.001	0.357	0.129–0.934	0.040
*g_Bacteroides*	0.290	0.134–0.608	0.004	0.357	0.129–0.935	0.040
*p_Firmicutes*						
*o_Gemellales*	1.710	0.834–3.548	0.145	4.209	1.527–12.911	0.008
*f_Gemellaceae*	1.710	0.834–3.548	0.291	4.209	1.527–12.911	0.015
*g_Bulleidia*	1.710	0.834–3.548	0.145	3.564	1.281–10.846	0.028
*g_Catonella*	0.386	0.182–0.800	0.017	0.278	0.010–0.733	0.034
*s_moorei*	1.710	0.834–3.548	0.145	3.564	1.281–10.846	0.037
**Rare microbiota**						
*p_Firmicutes*						
*g_Bacillus*	1.429	0.624–3.333	0.400	5.963	1.512–3.18	0.037
*p_Proteobacteria*						
*f_Alcaligenaceae*	0.475	0.217–1.015	0.057	0.276	0.09–0.788	0.038
*g_Moraxella*	1.541	0.732–3.285	0.342	3.016	1.108–8.860	0.048

*Adjusted by gender, age, smoke, residential district, season and tumour location

We conducted three stratified multivariate analyses in order to determine the effects of age, sampling season, and residential district on the outcomes of taxon abundance analysis. The stratified analysis by age indicated that the relative abundance of only the genus *Moraxella* was higher among drinkers younger than 60 years. However, the microbes whose relative abundance varied among the non-drinking and drinking patients older than 60 years were from the family Bacteroidaceae and its genus *Bacteroides*, order Gemellales and its family Gemellaceae, *Bulleidia*, and its species *B. moorei*, genus *Catonella*, and family Alcaligenaceae. (Table S1). On the other hand, the stratified analysis by sampling season demonstrated that the relative abundance of the family Alcaligenaceae, the genus *Bulleidia* and its species *B. moorei*, were significantly different in drinkers when sampled in winter or spring, while that of the genus *Catonella* decreased when sampled in summer or autumn (Table S2). Finally, the stratified analysis by resident district implicated that relative abundance of the family Bacteroidaceae and its genus *Bacteroides* were higher, and that of the genus *Bacillus* was lower in drinkers from Zhangzhou city. However, the relative abundance of the order Gemellales, its family Gemellaceae, the genus Bulleidia and its species *B. moorei*, genus *Catonella* significantly differed between non-drinkers and drinkers from other districts of the Fujian province (Table S3).

### Drinking-based trend analysis

**Fig. 4:**
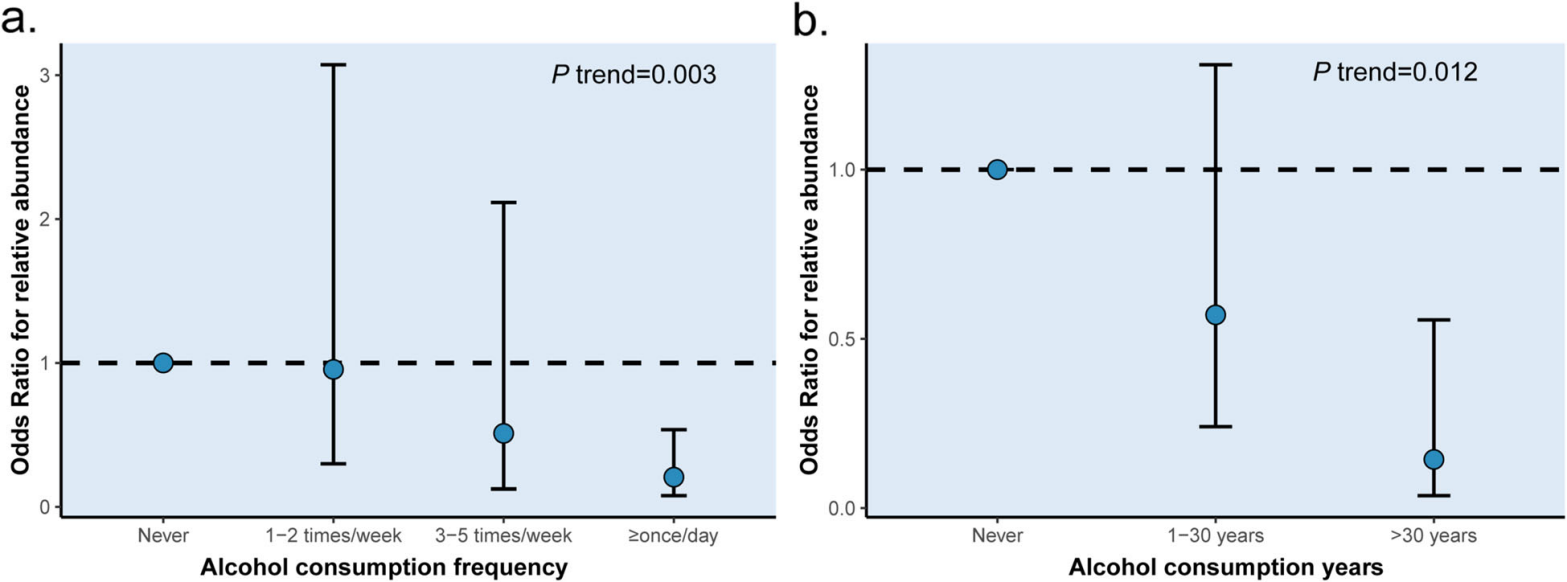
The trend between alcohol consumption and alterations of genus *Catonella.* (**a**. the trend for alcohol consumption frequency; **b**. the trend for alcohol consumption years)

We evaluated whether the relative abundance of alcohol-related bacteria was associated with frequency and years of alcohol consumption, and the taxon abundance was observed to vary with the frequency and years of alcohol consumption. The relative abundance of the genus *Catonella* decreased with increasing frequency and years of alcohol consumption (Table S4). The *OR* of the relative abundance of genus *Catonella* for drinking never, to more than once/day,was 1.000 (reference), 0.955 (95% *CI*:0.297–3.072), 0.509 (95% *CI*:0.123–2.115), and 0.204 (95% *CI*:0.078–0.535) (*P* = 0.003 for trend) (Fig. 4a). On the other hand, the *OR* of the relative abundance of genus *Catonella* from the lowest to highest years of alcohol consumption was 1.000 (reference), 0.571 (95% *CI*:0.240–1.309), and 0.143 (95% *CI*:0.037–0.556) (*P* = 0.012 for trend) (Fig. 4b).

## Discussion

Oesophageal cancer is a disease with high incidence and poor prognosis, which greatly threatens human health. Although alcohol consumption is assumed to contribute to the progress of malignant tumours, the exact molecular mechanism still remains unknown. Recently, human microbiota has been reported in many gastrointestinal diseases [14], including ESCC. An imbalance of microbiota may be an important indicator of the occurrence and development of ESCC [12]. Till date, the potential role of the oesophageal microbiota in drinking ESCC patients has not been investigated. We detected significant changes in the microbiota composition of oesophageal tissue between non-drinkers and drinkers with ESCC. Several specific bacteria were detected among the drinkers. Further analysis indicated that trends in the alteration of abundance of taxa became increasingly apparent as the frequency and years of alcohol consumption increased.

Host physiology and environmental factors can shape the microbiota diversity and composition [7]. Alcohol may be a possible modulator of the gastric microenvironment. In fact, alcohol exposure reportedly decreased intestinal bacterial diversity in mice [15]. Llopis [16] et al. identified significant differences in intestinal microbial communities between patients with severe alcoholic hepatitis and those with no alcoholic hepatitis. This phenomenon was also ascertained through animal gut microbiota trials. Fan et al. [17] reported significant difference in the diversity of oral microbiota and overall bacterial profiles among heavy drinkers and non-drinkers. Our results indicated that the Shannon diversity index and Bray-Curtis distance of oesophageal microbiota was varied remarkably even among the drinkers. Thus, it is evident that alcohol consumption significantly influences the microbiota composition, regardless of whatever it is oral, oesophageal, intestinal, or gut microbiota.

It was demonstrated in the present study that alcohol consumption is associated with an alteration in the abundance of certain bacteria. These bacteria belonged to the phyla Actinobacteria, Bacteroidetes, Firmicutes, and Proteobacteria. Our results were consistent with previous reports, where chronic alcohol use in humans was associated with an increased abundance of *Bacillus* and decrease in Bacteroidetes population in the colonic mucosa of a subset of alcoholics compared to the healthy controls [18]. Moreover, the growth of *Firmicutes* in gastrointestinal microbiota was reported to have significantly altered after alcohol consumption in healthy population, this alteration could affect the host’s metabolism [8]. In addition, bacteria of the phylum Bacteroides have been shown to decrease significantly in the gut microbiota of alcoholic patients [19]. In animal trials, the abundance of genera, including *Bacillus* and *Bacteroides* was changed remarkedly in ethanol-administered mice compared to that in mice on the normal diet [20]. Chronic alcohol consumption also caused a decline in *Firmicutes* and a proportional increase in *Actinobacteria* [15]. These studies are expected to help identify potential novel targets to prevent alcohol-associated pathologies.

The human microbiota is a changing ecosystem, continuously shaped by several factors, such as diet, season, lifestyle, districts or diseases [21]. Our stratified multivariate analysis also demonstrated the oesophageal microbiota in drinkers to be significantly affected by different age, residential districts and sampling seasons. A previous study [22] has indicated that gastrointestinal inflammation and permeability can be affected by alterations in the microbiota caused by age. Another recent study [23] revealed that some beneficial gut bacteria might be lost with age. Furthermore, Rampelli’s findings [24] demonstrated that the gut microbiome of the elderly population showed a rearrangement in metabolic pathways related to carbohydrate, amino acid, and lipid metabolism. Oakley and his colleagues [25] suggested that caecal microbial communities were greatly influenced by season of outgrowth. Thus, fewer microbiota were detected in winter than in spring or summer. Likewise, Sun [26] established that the composition and diversity of stool fungi varied significantly in mammals, depending on season. Davenport [27] et al. suggested that shifts in human microbiome composition can explain the influence of seasonal factors through dietary fluctuations. He [28] et al., in their study on the population of 14 districts of the Guangdong province in China, illustrated that host location was strongly associated with gut microbiota variations. An Iranian research team [11] demonstrated a relationship between the alterations in the gastric mucosal microbiota and the risk of ESCC carcinogenesis. In China, Firmicutes, Bacteroidetes and Proteobacteria are primary components of the oesophageal microbial environment in ESCC [12]. From these reports, it is evident that residential district has considerable effects on the human microbiome, which is also consistent with the observations from our study. Thus, all the previous studies cumulatively indicate that human microbiota can be influenced by either a single factor or interactions among multiple factors.

Decreased diversity and altered microbiota profiles in drinkers were observed in this study, which may be due to the direct effects of alcohol. Multiple studies [29, 30] have demonstrated that chronic alcohol consumption impaired gastrointestinal tract functions, leading to constant systemic inflammation and organ damage. Additionally, alcohol can contribute to microbial proliferation [31] and increased bacterial penetration by regulating inflammatory reaction [32]. *Moraxella* has been frequently shown to be associated with organ inflammation [33–35], which can explain the higher abundance of *Moraxella* in the oesophagus of the ESCC-affected drinkers of our study. In fact, the gastrointestinal mucosa may provide sites for the colonisation of pathogens. Mutlu [36] observed that daily alcohol administration to rats for 10 weeks led to gut dysbiosis, which may alter the gastrointestinal permeability, thereby exerting a direct deleterious effect on the mucosa [37]. Thus, damaged gastrointestinal mucosa plays a considerable role in the gradual progression of carcinogenesis.

Alcohol metabolites may also be indirectly responsible for microbiota alteration caused by alcohol consumption. It has been demonstrated that the impact of alcohol is modulated by enzymes associated with ethanol metabolism, including alcohol dehydrogenases and aldehyde dehydrogenases. The primary metabolite of alcohol —acetaldehyde, a plausible candidate involved in the carcinogenic process [38] — is further metabolised to acetic acid, and increased production of acetic acid has already been associated with lower relative abundance of Firmicutes, Proteobacteria, and Alcaligenaceae in the gut microbiota of mice [39]. In addition, *Moraxella* secretes an enzyme, TAE123, which can effectively oxidise alcohols [40, 41]. This can also explain why *Moraxella* is generally found in abundance among drinkers.

Additionally, alteration of luminal microenvironment in the oesophagus may modify the microbial communities. Our results showed a remarkable decrease in Alcaligenaceae and increase in *Moraxella,* which may account for chronic alcohol consumption, suggesting that alterations of the luminal microenvironment are possibly due to excessive acetic acid and decreased pH. Furthermore, luminal pH alteration may be a vital factor in the ethanol-induced shifts of intestinal microbiota [15]. pH exposure also changes the diversity of the intestinal bacteria, and disturbs the composition of the microbiota [42]. In addition, Chandel [43] reported that salivary pH increased significantly with decreasing abundance of *Moraxella*. Thus, it can be inferred that decreased oesophageal pH may lead to obvious proliferation of *Moraxella* after abundant alcohol consumption which is similar to our findings.

## Conclusion

This study investigated the alcohol-related microbiota in the oesophagus to demonstrate that alcohol consumption may be involved in the alteration of both the diversity and composition of oesophageal microbiota of ESCC patients. These alterations, among the drinkers, are affected by their age, residential districts, and even sampling seasons.

## Limitations

However, this study had several limitations. First, our experiment protocol lacked of steps to extract the DNA of Gram positive bacteria. This may overestimate the proportion of Gram negative bacteria in the results. It should be necessary to take some special measures to detect more Gram positive bacteria. In addition, about the only 120 cases of ESCC-related tissue microbiota were analysed in our study. A larger sample size should be involved to validate our findings. Indeed, the observed relationship may be regarded as a statistical association, owing to the cross-sectional nature of this study. Larger prospective cohort studies are also required to further determine the absolute relationship between alcohol consumption and oesophageal microbiota. Moreover, the biological mechanism by which alcohol influences the oesophageal microbiota still needs to be elucidated.

## Methods

### Participants details

We performed a hospital-based retrospective study of 120 patients pathologically diagnosed with primary ESCC between February 2013 and October 2017 at Fujian Provincial Cancer Hospital and Zhangzhou Municipal Hospital. Subjects were chosen according to the following criteria. Inclusion criteria: (a) underwent oesophagectomy surgery; (b) pathologically diagnosed with primary ESCC; (c) tumour stage clarified with number of dissected lymph nodes ≥20; (d) undergoing neither radiotherapy nor chemotherapy; (e) no antibiotic use through preoperative 2 months; (g) no record of other infectious diseases; and (h) resident of Fujian province for more than 10 years. Exclusion criteria: (a) incomplete clinicopathological data and non-availability of tissue samples; (b) metastatic malignancy or recurrent oesophageal cancer; (c) received pharmacotherapy (such as oral, intramuscular, and intravenous antibacterial drugs, various probiotics or other drugs affecting the microbiota, and thereby influencing the trail result) within a month. Written informed consent was obtained from all the patients. The study was approved by the Ethics Committee of Fujian Medical University (approval no. 201495).

### Demographic information

The basic information of all the participants was collected through a detailed questionnaire comprising of sociodemographic status, dietary habits, daily physical activity, smoking status, alcohol consumption, family history of cancer and gastrointestinal symptoms. Clinicopathological features (viz. differentiation status, location, and tumour, node, and metastasis (TNM) stage) for each patient were also collected from their respective medical records. Smoking status was defined as smoking at least one cigarette per day continuously for at least 6 months [44, 45]. Alcohol consumption status was defined by consumption of alcohol at least once a week, with an alcohol intake of ≥50 g per time for six consecutive months [46–48], depending on which, patients were divided into drinkers and non-drinkers. Frequency (Never, 1-2times/week, 3-5times/week, ≥once/day) and years (Never, 1-30 years, ≥30 years) of alcohol consumption were also recorded for the drinkers. Tea consumption was defined as having at least one cup per week for 6 months or more [49, 50].

### Tissue specimen collection and preservation

Tissue samples were obtained from each patient immediately after surgical resection in the operating room, from an area at a distance of 3 cm from the cancerous tissue. The adjacent tissues samples were cut into small pieces and placed in autoclaved cryovials, stored in liquid nitrogen for 1 h, and, then transferred to a − 80 °C refrigerator for storage. No tumour cells were detected in any adjacent tissues by pathological haematoxylin-eosin (HE) staining.

### Bacterial DNA extraction and 16S rRNA sequencing

The sodium dodecyl sulphate (SDS) method was used to extract bacterial DNA from the specimens. The extracted DNA was quantitatively detected by Qubit fluorometer (Invitrogen, America), and the results were acceptable. Each extraction was performed with a blank buffer control to detect contaminants from either reagents, or other unintentional sources. However, the negative controls detected too few DNA to prepare library and hence were not sequenced.

Amplification of the 16S rRNA gene used primers targeting regions V3–V4, which included forward primer (341F: 5′-CCTAYGGGRBGCASCAG-3′) and reverse primer (806R: 5′-GGACTACNNGGGTATCTAAT-3′). The sequencing platform was the HiSeq2500 PE250(Illumina, America).

### Sequence data processing

**Fig. 5:**
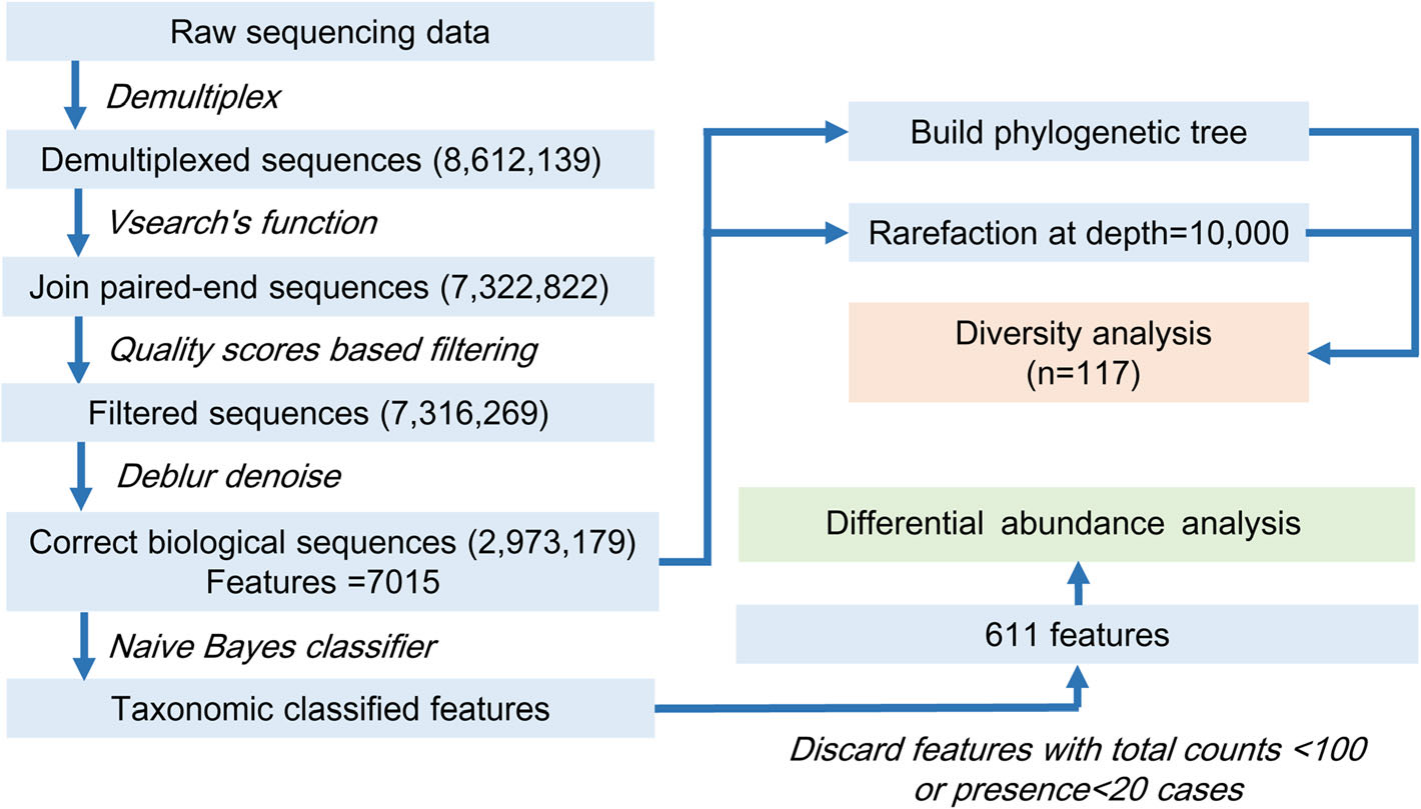
The analysis flowchart of DEBLUR algorithm using QIIME2

Raw sequencing data from patients with ESCC were imported into Quantitative Insights Into Microbial Ecology (QIIME2–2019.04) [51] and processed using the DEBLUR algorithm to denoise and then inferred exact amplicon sequence variants (ASVs) (Fig. 5). The curated ASVs were aligned and annotated by the Naïve Bayes classifier using the Greengenes (version 13.5) database, and were used for the subsequent construction of the phylogenetic tree. In alpha and beta diversity analysis, the resampling depth was set at 10,000 reads to ensure sufficient reads and sample size. Alpha diversity was evaluated against species evenness, Shannon diversity, Faith’s phylogenetic diversity and observed amplicon sequence variants (ASVs). Beta-diversity was calculated by Bray-Curtis distance, Jaccard distance, and both weighted and unweighted UniFrac distances. Linear discriminant analysis effect size (LEfSe) [52] and Unconditional logistic regression were used to determine which bacteria were associated with alcohol consumption. Bacteria with sequencing counts of more than 100, and those detected in at least 20 specimens were designated as the reserved species in order to reduce the effect of excessive species on the false discovery rate (FDR). Dominant microbiota was defined as the relative abundance of bacteria over 0.01%. The others were considered rare microbiota.

### Statistical analysis

Questionnaires and clinicopathological data were double-entered into EpiData (version 3.1, Denmark). The relationships between demographic and baseline clinical features and alcohol consumption were evaluated using the Chi-square test. The odds ratio (*OR*) and 95% confidence interval (*CI*) were estimated using unconditional logistic regression. All statistical analyses were evaluated using SPSS (v19.0, Chicago, USA), and two-tailed *P* < 0.050 was considered statistically significant. *P* values were adjusted by false discovery rate (FDR) correction according to the Benjamini Hochbergs procedure.

## Supplementary Information

**Additional file 1: Table S1.** Stratified multivariate analysis by age (p:phylum, o:order,f:family, g:genus, s:species). **Table S2.** Stratified multivariate analysis by sampling season (p:phylum, o:order,f:family, g:genus, s:species). **Table S3.** Stratified multivariate analysis by residential district (p:phylum, o:order,f:family, g:genus, s:species). **Table S4.** Drinking-based trend analysis. **Figure S1.** Linear discriminant analysis effect size identified thirteen bacterial taxa which were significantly different between the two groups.

## Data Availability

Data are available in a public, open access repository. The authors confirm all supporting data have been provided in NCBI BioProject (Accession number: PRJDB10075) (https://www.ncbi.nlm.nih.gov/bioproject/?term=PRJDB10075).

## Abbreviations

ESCC: Oesophageal squamous cell carcinoma
QIIME: Quantitative insights into microbial ecology
LEfSe: Linear discriminant analysis effect size
EC: Oesophageal cancer
ASVs: Amplicon sequence variants
FDR: False discovery rate
OR: Odds ratio
CI: Confidence interval
