# Supplementary material for: Association between alcohol consumption and oesophageal microbiota in oesophageal squamous cell carcinoma
Source: BMC Microbiol. 2021 Mar 6;21:73. doi: 10.1186/s12866-021-02137-x (PMC7936487; doi:10.1186/s12866-021-02137-x)
Supplement: Supplementary file 1 — Additional file 1: Table S1. Stratified multivariate analysis by age (p:phylum, o:order,f:family, g:genus, s:species). Table S2. Stratified multivariate analysis by sampling season (p:phylum, o:order,f:family, g:genus, s:species). Table S3. Stratified multivariate analysis by residential district (p:phylum, o:order,f:family, g:genus, s:species). Table S4. Drinking-based trend analysis. Figure S1. Linear discriminant analysis effect size identified thirteen bacterial taxa which were significantly different between the two groups. [file 12866_2021_2137_MOESM1_ESM.docx]

**Association between alcohol consumption and oesophageal microbiota in oesophageal squamous cell carcinoma**

**Wenqing Rao^1^, Zheng Lin^1^, Shuang Liu^1^, Zhihui Zhang^1^, Qianwen Xie^1^, Huilin Chen^2^, Xi Lin^3^, Yuanmei Chen^4^, Huimin Yang^1^, Kaili Yu^1^, Zhijian Hu^1,5^***

^1^Department of Epidemiology and Health Statistics, Fujian Medical University Fujian Provincial Key Laboratory of Environment Factors and Cancer, School of Public Health, Fujian Medical University, Fuzhou 350122, China.

^2^Department of Radiation Oncology, Anxi County Hospital, Quanzhou 352400, China

^3^Department of Statistics Office, Zhangzhou Affiliated Hospital of Fujian Medical University, Zhangzhou 363000, China.

^4^Department of Thoracic Surgery, Fujian Provincial Cancer Hospital Affiliation to Fujian Medical University, Fuzhou 350014, China.

^5^Key Laboratory of Ministry of Education for Gastrointestinal Cancer, Fujian Medical University, Fuzhou 350122, China.

The subject category that applied to the manuscript: Research article.

***Corresponding author:** Zhijian Hu, Department of Epidemiology and Health Statistics, Fujian Provincial Key Laboratory of Environment Factors and Cancer, School of Public Health, Key Laboratory of Ministry of Education for Gastrointestinal Cancer, Fujian Medical University, Fuzhou 350122, China.

E-mail address: [huzhijian@fjmu.edu.cn](mailto:huzhijian@fjmu.edu.cn)

Telephone: 0086-591-83383362

Fax: 0086-591-822862510

**Table S1**. Stratified multivariate analysis by age

（p:phylum, o:order,f:family, g:genus, s:species）

| Bacteria | **Age≤60(n=56)** | | |  | **Age>60(n=64)** | | |
| --- | --- | --- | --- | --- | --- | --- | --- |
|  | *OR* | 95*%CI* | *P* |  | *OR* | 95*%CI* | *P* |
| **Dominant microbiota** |  |  |  |  |  |  |  |
| *p_Actinobacteria* |  |  |  |  |  |  |  |
| *s_longum* | 6.232 | 1.002-50.117 | 0.184 |  | 2.121 | 0.569-8.478 | 0.269 |
| *p_Bacteroidetes* |  |  |  |  |  |  |  |
| *f_Bacteroidaceae* | 0.331 | 0.051-1.864 | 0.438 |  | 0.267 | 0.061-1.019 | 0.062 |
| *g_Bacteroides* | 0.331 | 0.051-1.864 | 0.328 |  | 0.267 | 0.061-1.019 | 0.062 |
| *p_Firmicutes* |  |  |  |  |  |  |  |
| *o_Gemellales* | 2.611 | 0.467-17.748 | 0.288 |  | 7.160 | 1.780-37.349 | 0.009 |
| *f_Gemellaceae* | 3.611 | 0.467-17.749 | 0.288 |  | 7.160 | 1.780-37.349 | 0.018 |
| *g_Bulleidia* | 1.708 | 0.322-10.152 | 0.535 |  | 6.566 | 1.560-33.185 | 0.020 |
| *g_Catonella* | 0.299 | 0.042-1.750 | 0.590 |  | 0.130 | 0.028-0.503 | 0.015 |
| *s_moorei* | 1.708 | 0.322-10.152 | 0.642 |  | 6.566 | 1.560-33.185 | 0.040 |
| **Rare microbiota** |  |  |  |  |  |  |  |
| *p_Firmicutes* |  |  |  |  |  |  |  |
| *g_Bacillus* | 17.576 | 1.621-520.055 | 0.079 |  | 3.269 | 0.556-29.187 | 0.448 |
| *p_Proteobacteria* |  |  |  |  |  |  |  |
| *f_Alcaligenaceae* | 1.006 | 0.178-5.888 | 0.995 |  | 0.097 | 0.016-0.447 | 0.011 |
| *g_Moraxella* | 57.918 | 4.899-2196.596 | 0.028 |  | 1.384 | 0.400-4.938 | 0.811 |

**Table S2**. Stratified multivariate analysis by sampling season

（p:phylum, o:order,f:family, g:genus, s:species）

| Bacteria | **Winter/Spring(n=70)** | | |  | **Summer/Autumn(n=50)** | | |
| --- | --- | --- | --- | --- | --- | --- | --- |
|  | *OR* | 95*%CI* | *P* |  | *OR* | 95*%CI* | *P* |
| **Dominant microbiota** |  |  |  |  |  |  |  |
| *p_Actinobacteria* |  |  |  |  |  |  |  |
| *s_longum* | 1.137 | 0.274-4.619 | 0.857 |  | 9.403 | 1.437-95.860 | 0.093 |
| *p_Bacteroidetes* |  |  |  |  |  |  |  |
| *f_Bacteroidaceae* | 0.275 | 0.065-1.040 | 0.065 |  | 0.2 | 0.003-1.163 | 0.168 |
| *g_Bacteroides* | 0.275 | 0.065-1.041 | 0.097 |  | 0.2 | 0.003-1.163 | 0.126 |
| *p_Firmicutes* |  |  |  |  |  |  |  |
| *o_Gemellales* | 3.365 | 0.967-12.937 | 0.064 |  | 7.172 | 0.921-157.875 | 0.103 |
| *f_Gemellaceae* | 3.365 | 0.967-12.937 | 0.127 |  | 7.172 | 0.671-76.633 | 0.103 |
| *g_Bulleidia* | 11.166 | 2.585-62.113 | 0.008 |  | 1.112 | 0.196-7.074 | 0.905 |
| *g_Catonella* | 0.579 | 0.150-2.169 | 0.418 |  | 0.026 | 0.001-0.232 | 0.012 |
| *s_moorei* | 11.166 | 2.585-62.113 | 0.015 |  | 1.112 | 0.196-7.074 | 1.000 |
| **Rare microbiota** |  |  |  |  |  |  |  |
| *p_Firmicutes* |  |  |  |  |  |  |  |
| *g_Bacillus* | 6.049 | 0.948-60.608 | 0.160 |  | 13.594 | 1.211-424.045 | 0.134 |
| *p_Proteobacteria* |  |  |  |  |  |  |  |
| *f_Alcaligenaceae* | 0.097 | 0.002-0.444 | 0.009 |  | 2.209 | 0.362-15.061 | 0.393 |
| *g_Moraxella* | 1.982 | 0.582-7.117 | 0.373 |  | 18.65 | 1.923-638.274 | 0.136 |

**Table S3**. Stratified multivariate analysis by residential district

（p:phylum, o:order,f:family, g:genus, s:species）

| Bacteria | **Zhangzhou(n=50)** | | |  | **Others(n=70)** | | |
| --- | --- | --- | --- | --- | --- | --- | --- |
|  | *OR* | 95*%CI* | *P* |  | *OR* | 95*%CI* | *P* |
| **Dominant microbiota** |  |  |  |  |  |  |  |
| *p_Actinobacteria* |  |  |  |  |  |  |  |
| *s_longum* | 1.647 | 0.363-7.718 | 0.773 |  | 11.345 | 1.680-232.487 | 0.052 |
| *p_Bacteroidetes* |  |  |  |  |  |  |  |
| *f_Bacteroidaceae* | 0.008 | 0.008-49.801 | 0.020 |  | 0.855 | 0.205-3.659 | 0.828 |
| *g_Bacteroides* | 0.008 | 0.008-49.801 | 0.030 |  | 0.855 | 0.205-3.659 | 0.828 |
| *p_Firmicutes* |  |  |  |  |  |  |  |
| *o_Gemellales* | 2.474 | 0.608-11.073 | 0.215 |  | 28.870 | 3.798-418.035 | 0.004 |
| *f_Gemellaceae* | 2.474 | 0.608-11.073 | 0.215 |  | 28.870 | 3.798-418.035 | 0.009 |
| *g_Bulleidia* | 5.609 | 1.209-33.659 | 0.057 |  | 2.872 | 0.631-15.759 | 0.284 |
| *g_Catonella* | 0.534 | 0.113-2.364 | 0.413 |  | 0.097 | 0.016-0.459 | 0.018 |
| *s_moorei* | 5.609 | 1.209-33.659 | 0.226 |  | 2.872 | 0.631-15.759 | 0.228 |
| **Rare microbiota** |  |  |  |  |  |  |  |
| *p_Firmicutes* |  |  |  |  |  |  |  |
| *g_Bacillus* | 18.750 | 2.709-388.872 | 0.046 |  | 1.065 | 0.131-1.114 | 0.945 |
| *p_Proteobacteria* |  |  |  |  |  |  |  |
| *f_Alcaligenaceae* | 0.330 | 0.061-1.518 | 0.337 |  | 0.199 | 0.033-1.053 | 0.063 |
| *g_Moraxella* | 1.664 | 0.366-8.236 | 0.687 |  | 21.950 | 2.859-306.258 | 0.033 |

**Table S4.** Drinking-based trend analysis

| **Drinking** | **g_*Catonella*** | |
| --- | --- | --- |
| **Frequency** | ***OR*(95%*CI*)** | ***P* for trend** |
| Never | 1.000 | 0.003 |
| 1-2 times/week | 0.955(0.297-3.072) |  |
| 3-5 times/week | 0.509(0.123-2.115) |  |
| ≥once/day | 0.204(0.078-0.535) |  |
| **Years** |  |  |
| 0 | 1.000 | 0.012 |
| 0-30 | 0.571(0.240-1.309) |  |
| 30-60 | 0.143(0.037-0.556) |  |


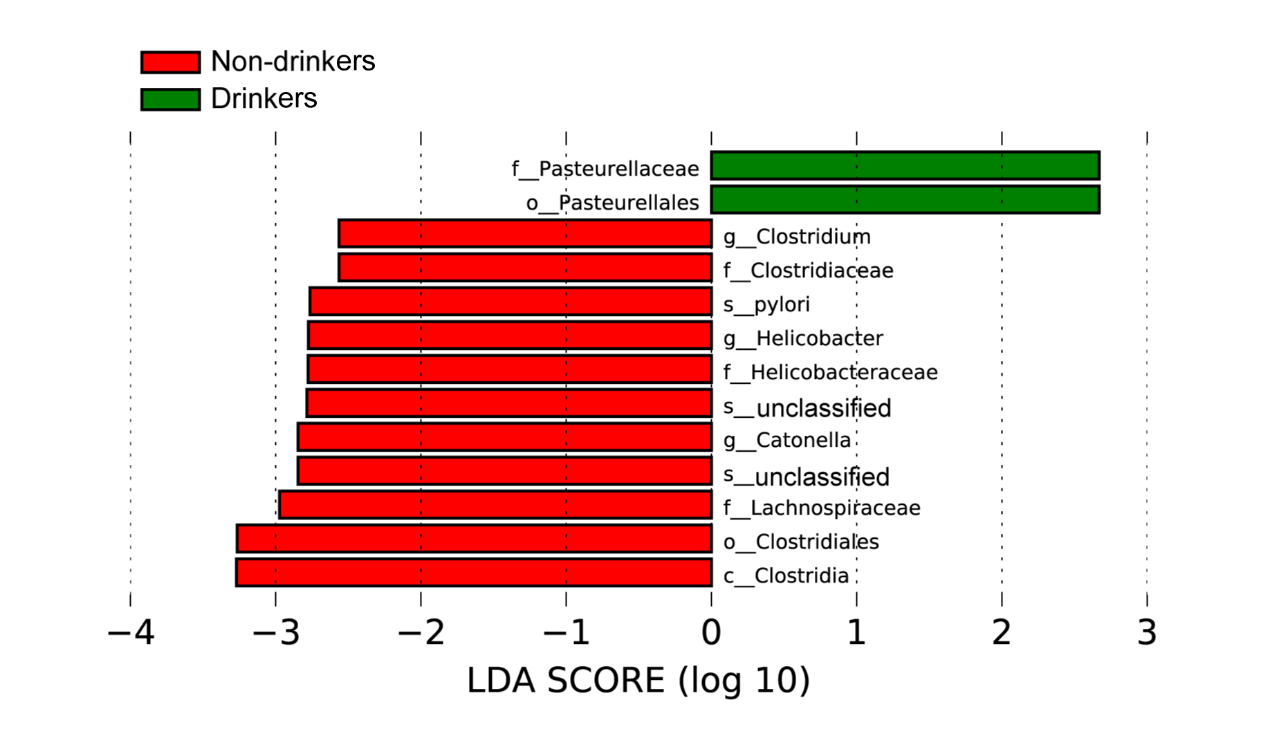


**Figure S1.** Linear discriminant analysis effect size identified thirteen bacterial taxa which were significantly different between the two groups
